# Exploiting Surface Plasmon Resonance (SPR) Technology for the Identification of Fibroblast Growth Factor-2 (FGF2) Antagonists Endowed with Antiangiogenic Activity

**DOI:** 10.3390/s90806471

**Published:** 2009-08-20

**Authors:** Marco Rusnati, Antonella Bugatti, Stefania Mitola, Daria Leali, Paolo Bergese, Laura E. Depero, Marco Presta

**Affiliations:** 1 Unit of General Pathology and Immunology, Department of Biomedical Sciences and Biotechnology, School of Medicine, University of Brescia, Brescia, 25123, Italy; E-Mails: rusnati@med.unibs.it (M.R.); bugatti@med.unibs.it (A.B.); mitola@med.unibs.it (S.M.); leali@med.unibs.it (D.L.);; 2 Chemistry for Technologies Laboratory and Department of Mechanical and Industrial Engineering, School of Engineering, University of Brescia, Brescia, 25123, Italy; E-Mails: paolo.bergese@ing.unibs.it (P.B.); depero@ing.unibs.it (L.E.D.)

**Keywords:** angiogenesis, fibroblast growth factor-2, heparan-sulfate proteoglycans, heparin, interactions, pentraxin 3, peptides, surface plasmon resonance, thrombospondin-1

## Abstract

Angiogenesis, the process of new blood vessel formation, is implicated in various physiological/pathological conditions, including embryonic development, inflammation and tumor growth. Fibroblast growth factor-2 (FGF2) is a heparin-binding angiogenic growth factor involved in various physiopathological processes, including tumor neovascularization. Accordingly, FGF2 is considered a target for antiangiogenic therapies. Thus, numerous natural/synthetic compounds have been tested for their capacity to bind and sequester FGF2 in the extracellular environment preventing its interaction with cellular receptors. We have exploited surface plasmon resonance (SPR) technique in search for antiangiogenic FGF2 binders/antagonists. In this review we will summarize our experience in SPR-based angiogenesis research, with the aim to validate SPR as a first line screening for the identification of antiangiogenic compounds.

## 1. The Angiogenesis Process

Angiogenesis, the process of new blood vessel formation from pre-existing ones, plays a key role in various physiological and pathological conditions, including embryonic development, wound repair, inflammation, and tumor growth [1]. The local, uncontrolled release of angiogenic growth factors (AGFs) and/or alterations of the production of natural angiogenic inhibitors, with a consequent alteration of the angiogenic balance, are responsible for the uncontrolled endothelial cell (EC) proliferation that takes place during tumor neovascularization and angiogenesis-dependent diseases [2].

The 1980s saw for the first time the purification to homogeneity of pro-angiogenic proteins, the breakthrough coming as a result of the observation that EC growth factors showed a marked affinity for heparin [3,4]. This lead to the identification, purification, and sequencing of the two prototypic heparin-binding angiogenic fibroblast growth factors (FGF) 1 and 2. Since then, numerous inducers of angiogenesis have been identified, including the members of the vascular endothelial growth factor (VEGF) family, angiopoietins, transforming growth factor-α and -β (TGF-α and TGF-β), platelet-derived growth factor (PDGF), hepatocyte growth factor (HGF), tumor necrosis factor-α (TNF-α), interleukins (ILs), and chemokines [5].

**Figure 1.**
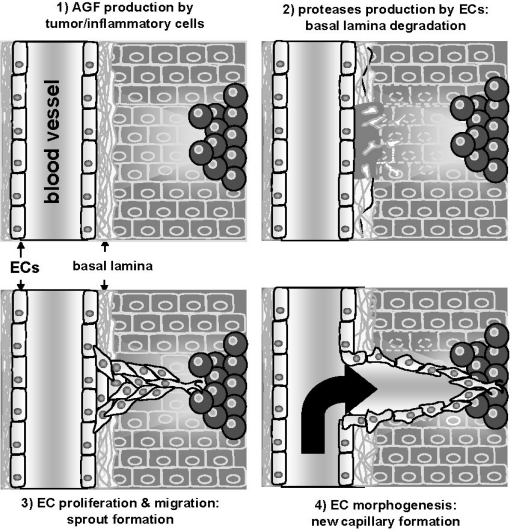
Schematic representation of the neovascularization process. Adapted from [14].

Angiogenesis is a multi-step process that begins with the degradation of the basement membrane by activated ECs that will then migrate and proliferate, leading to the formation of solid EC sprouts into the stromal space. Later, EC sprouts undergo a morpho-functional rearrangement, giving origin to a functional capillary (Figure 1).

Accordingly, AGFs induce a complex “pro-angiogenic phenotype” in cultured ECs that recapitulates the neovascularization process, including production of proteases that degrade the extracellular matrix (ECM), EC migration, and proliferation. These two latter biological processes, that lead to the formation of endothelial sprouts, are tightly regulated by lateral cell-cell adhesion and ECM interactions [6] that, in turn, are mediated by cadherin and integrin receptors. Accordingly, AGFs regulate EC adhesion (reviewed in [7]), and the expression of different integrins [8] and cadherins [6] in a complex fashion. Indeed, a brief exposure to the angiogenic FGF2 hampers endothelial cell-cell junctions whereas a prolonged exposure to the growth factor promotes a slow temporal re-distribution of junctional adhesion proteins, suggesting that AGFs promotes both EC scattering, required during the first steps of angiogenesis, and the formation of the cell-cell interactions necessary to vessel maturation [6]. A similar biphasic regulation exists also for the composition of the ECM that surrounds the endothelium: AGFs initially promote the disruption of the basal lamina and lately induce the production of appropriate ECM components by ECs [9]. The final step in angiogenesis consists in the so called “morphogenesis” process characterized by the organization of ECs in “capillary-like structures” that will mature in functional vessels. Again, this process requires the activation of the proteolytic machinery [10],□ integrins [11], and junctional adhesion molecules [12] that, in turn, are controlled by the activity of AGFs and their tyrosine kinase (TK)-receptors [13].

## 2. Surface Plasmon Resonance (SPR) Spectroscopy for the Study of the Angiogenesis Process

**Figure 2.**
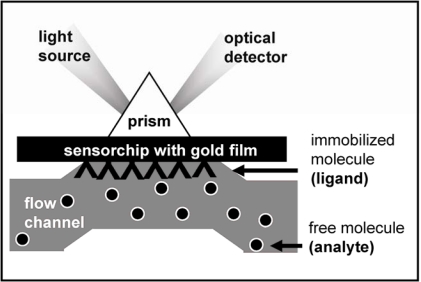
Schematic representation of SPR technology. The molecule immobilized onto the gold film of the sensor chip is named ligand whereas the analyte is represented by the putative partner injected into the microfluidic system.

A typical setup of a solid-phase bioassay based on SPR spectroscopy is shown in Figure 2. Polarized beam of monochromatic visible light is passed through a prism fitted with a glass slide coated with about 50 nm of gold (or other metal). The light is reflected off the gold and its intensity is detected at the specular angle. An electric field intensity, known as an evanescent wave, is generated when the light strikes the glass. This evanescent wave interacts with, and is absorbed by, free electron clouds in the gold layer, generating electron charge density waves called plasmons and causing a reduction in the intensity of the reflected light. The angle corresponding to the sharp intensity minimum that occurs at the SPR condition is called resonance angle. It depends on the refractive index (RI) of the material above and near (below 300 nm) the gold surface, as it is sampled by the evanescent light intensity. The resonance angle can be monitored by following the specularly reflected light intensity *versus* angle at fixed wavelengths or *versus* wavelength at fixed angle. In a SPR assay, the receptor specific for a particular analyte is chemically immobilized onto the gold film. When the sensor is exposed to a sample containing that analyte, the binding of the analyte to the receptor causes a change of the RI at the metal surface resulting in the shift of the resonance angle and providing label-free transduction of the binding reaction. Due to its peculiar architecture, SPR bioassays add to label-free molecular recognition several advantages, including the ability to investigate and manipulate minute concentrations of molecules semi-automatically, in real time and multiplexed way and the access to information spanning from straight ON-OFF sensing to binding thermodynamics and kinetics. For these reasons SPR represents a powerful asset in the study of biomolecular interactions, including the molecular bases of angiogenesis [15–23].

As mentioned above, AGFs establish a network of extracellular interactions in order to exert their full angiogenic potential. Indeed, a complex molecular “interactome” due to the cross-talk among cell surface receptors, ECM components, and free molecules appears to modulate the angiogenic balance in normal and pathological settings [7]. In this context, SPR has been usefully exploited to demonstrate and/or characterize the binding of AGFs with their interactors, including cell surface signalling receptors (Table 1) and extracellular proteoglycans (Table 2).

From the analysis of these tables it emerges that the binding parameters may vary significantly not only among different binding partners but also for the same interactors in different studies. These discrepancies may be due to suboptimal data and/or over-interpreted results [15–23]; different immobilization procedures of the ligand; the use of different molecular forms of the ligand and/or of the analyte (e.g., different receptor isoforms, different AGF variants, glycosaminoglycans (GAGs) of different chemical structure). See also Section 5 for a further discussion about this point.

Beside the characterization of the direct AGF/receptor interaction, SPR has been also employed to measure the concentration of AGFs in body fluids. This has been successfully achieved for HGF [55–57] and has been proposed also for FGF2 [58].

Finally, different SPR-based experimental models have been set up and used for the identification of antiangiogenic compounds (Table 3). With these models, various promising inhibitors (including neutralizing antibodies directed against AGFs or their receptors) have been identified and characterized for their molecular mechanism(s) of action (see references in Table 3).

## 3. Fibroblast Growth Factor-2 (FGF2) and Its Receptors

FGF2 is a pleiotropic factor able to stimulate different cell types, including ECs, by interacting with specific tyrosine kinase (TK) receptors (FGFRs). The four members of the FGFR family [FGFR1 (*flg*), FGFR2 (*bek*), FGFR3 and FGFR4] are encoded by distinct genes and their structural variability is increased by alternative splicing of their RNA transcripts [99]. The extracellular portion of FGFRs comprises three Ig-like domains (D1, D2, and D3, with an acidic box between D1 and D2). Their ligand binding and specificity reside in D2, D3, and D2–D3 linker region. X-ray crystallography has shown that the interactions between FGF2 and D3 are of both hydrophobic and polar character whereas the interactions with the D2–D3 linker are mediated mainly via hydrogen bonds. At variance, hydrophobic interactions dominate the interface between FGF2 and D2 [100].

FGFR1 is expressed by ECs *in vivo* [101]. *In vitro*, ECs of different origin express FGFR1 [102] and, under some circumstances, FGFR2 [103], whereas the expression of FGFR3 or FGFR4 has never been reported in endothelium. The interactions of FGFs with FGFRs occur with high affinity [dissociation constant (K_d_) = 10–550 pM] and trigger the activation of complex signal transduction pathways [104]. However it must be pointed out that, to induce a full angiogenic response, FGF2 needs also to interact with integrin α_v_β_3_ [105], ganglioside GM1 [106] and HSPGs (discussed below).

HSPGs typically consist of a core protein and of GAG chains. HSPGs are found associated to the surface of almost all eukariotic cells, including ECs, at concentrations ranging between 10^5^–10^6^ molecules/cell. Noticeably, HSPG expression in ECs derived from the microvasculature (where the angiogenic process originates) is 10–15 times higher than that found in macrovascular ECs, supporting the role of HSPGs in neovascularization [107]. HSPGs can link to the plasma membrane through a hydrophobic transmembrane domain of their core protein or through a glycosyl-phosphatidylinositol (GPI) anchor covalently bound to the core protein. Typical transmembrane HSPGs are syndecans, the most represented transmembrane HSPGs in ECs [108]. Glypicans and cerebroglycan are typical GPI-anchored HSPGs. Perlecan is instead a typical peripheral membrane HSPG [109].

HSPGs present at the basal site of blood vessels act as receptors for basement membrane proteins. HSPGs present at the luminal surface of ECs [110] contribute to the anticoagulative properties of the vessel surface by binding proteases of the intrinsic coagulation cascade [111] and act as receptors or co-receptors for a variety of cytokines and growth factors, including FGF2. Usually the binding of these proteins to HSPGs occurs via their GAG-chains (discussed below).

**Figure 3.**
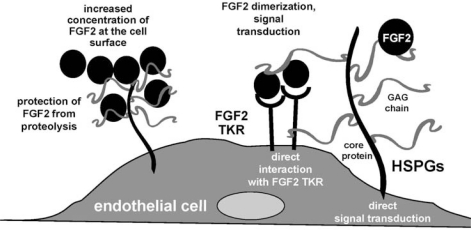
Schematic representation of the biological functions of HSPGs in FGF2 biology.

HSPGs exist also in free form following their mobilization from cell-surface or ECMs by proteolytic digestion of their core protein (for transmembrane HSPGs) or by the action of phospholipase (for GPI-anchored HSPGs). Also, free GAGs can be generated by enzymatic digestion of HSPGs GAG-chain (reviewed in [112]). The biological consequences of HSPGs/FGF2 interaction are manifold: FGF2 binding to EC-surface HSPG promotes angiogenesis *in vitro* and *in vivo* [113] by direct activation of intracellular signalling [113], by mediating FGF2 internalization [114], and/or by presenting FGF2 to FGFRs in a proper conformation [112]. Also, ECM-associated HSPGs act as a reservoir for FGF2 that is protected from degradation [115] and accumulates in the microenvironment to sustain a long-term stimulation of ECs [116] (Figure 3).

GAGs are negatively charged polysaccharides composed of repeating disaccharide units whose prototype is heparin. Heparin is a natural polysaccharide produced by mast cells. Once released, it regulates coagulation through the binding to coagulation factors such as antithrombin III and heparin cofactor II [117]. Also, like HSPGs, heparin binds to a variety of enzymes, cytokines and growth factors, including FGF2 [118]. This capacity, that depends on distinct chemical properties of the polysaccharide chains, can be exploited to design heparin-like drugs for pharmacological interventions in a variety of pathologic conditions including thrombosis, neoplasia and viral infection [94].

The interaction of heparin/HSPGs with FGF2 occurs with a K_d_ equal to 2–200 nM. Both heparin and GAGs/HSPGs from ECs bind FGF2 and protect it from inactivation and proteolytic degradation [119,120]. Also, free GAGs favour the delivery of FGF2 to the blood supply to stimulate angiogenesis by increasing the radius of diffusion of the growth factor [121]. Depending on its concentration, free heparin can act as a FGF2 agonist, inducing oligomerization of FGF2 [122] that is required for its full biological response [123], or as a FGF2 antagonist, sequestering FGF2 in the extracellular environment, hampering its interaction with ECs and inhibiting its biological activity [115].

**Figure 4.**
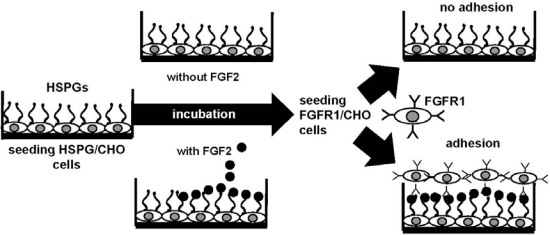
Schematic representation of the FGF2-mediated cell-cell adhesion model. HSPG-bearing CHO (HSPG/CHO) cells are seeded, allowed to reach confluence and incubated with or without FGF2. Next, FGFR1-bearing CHO (FGFR1/CHO) cells are incubated onto the HSPG/CHO cell monolayers. Finally, FGFR1/CHO cells adherent to the HSPG/CHO cell monolayers are counted under the microscope.

Heparin and HSPGs can interact also with FGFRs. Indeed, heparin/HSPGs, FGF2 and FGFR1 form a ternary complex in which the GAG chain interacts with both FGF2 and FGFR [124]. The formation of the HSPG/FGF2/FGFR1 ternary complex plays a central role in the biology of FGF2 and in the process of neovascularization. For this reason, it has been considered as a model for the development of angiogenesis-related assays and a target for the development of antiangiogenic compounds. To this purpose, a FGF2-dependent cell-cell adhesion (CCA) assay has been developed [125] and successfully exploited [94,126,127] for the identification of compounds able to disrupt the HSPG/FGF2/FGFR1 ternary complex, thus acting as angiogenesis inhibitors (Figure 4).

In this model, FGF2 mediates the interaction of HSPG-deficient CHO cells overexpressing FGFR1 to a monolayer of CHO cells expressing HSPGs but not FGFRs [128]. As a consequence of its capacity to interact with any of the three molecular partners involved in the interaction, the putative inhibitor is anticipated to prevent the formation of the HSPG/FGF2/FGFR1 ternary complex resulting from the simultaneous binding of FGF2 to HSPGs and FGFR1 expressed on neighbouring cells.

## 4. Extracellular FGF2 Antagonists: Exploiting SPR for the Identification of Antiangiogenic Compounds

FGF2 interacts with several partners that modulate its bioavailability, stability, local concentration and interaction with EC [129]. On the other hand, several molecules of the body fluids or of the ECM interact with the various FGF2 receptors expressed on the surface of ECs. The identification of these molecules and the characterization of their interactions with FGF2 or with FGF2 receptors may allow the design of efficient and specific inhibitors. Two main categories of FGF2-antagonists have been to date identified: proteins of the body fluids/ECM and related peptides; polyanionic, heparin-like compounds (for a review see [130]). The current approaches for the identification of FGF2 antagonists endowed with antiangiogenic capacity are based on the screening of large libraries of compounds. However, the available *in vitro* and *in vivo* experimental models for testing these compounds may be quite complex, time-consuming and expensive, calling for additional models overcoming these drawbacks. SPR assays may fit these requirements, being handy-user, reliable, and high-throughput.

### 4.1. Anti-FGF2 Peptides

Several ECM components, their degradation products or related synthetic peptides affect FGF-driven angiogenesis. Thrombospondins (TSP) are modular glycoprotein secreted by ECs that bind to HSPGs and integrin receptors [131]. TSP-1 exerts an antiangiogenic effect that is due, at least in part, to its capacity to bind FGF2 [132], preventing its interaction with HSPGs and FGFRs and inhibiting its mitogenic and chemotactic activity on ECs. TSP-1 also prevents FGF2 accumulation in the ECM and favors matrix-bound FGF2 mobilization, generating inactive TSP-1/FGF2 complexes [133]. Fibstatin is a fibronectin fragment that binds FGF2 and inhibits FGF2-dependent proliferation, migration and tubulogenesis of ECs *in vitro* and angiogenesis and tumor growth *in vivo* [134].

A variety of serum components can bind and regulate FGF2. α_2_-Macroglobulin (α_2_M) is a plasma protein that acts as a protease inhibitor and binds a variety of cytokines and growth factors [135]. α_2_M sequesters FGF2 in the extracellular environment inhibiting its cell interaction, protease-inducing activity [136], and mitogenic capacity [135]. Pentraxin 3 (PTX3) is a soluble pattern recognition receptor with unique functions in various physio-pathological conditions released by mononuclear phagocytes and ECs [137]. Its functions relay on its capacity to bind different molecules, including FGF2 [138]. PTX3 prevents the binding of FGF2 to EC surface FGFRs, inhibits EC proliferation and migration, and FGF2-dependent neovascularization and tumorigenesis *in vivo* [138]. PDGF-BB [139], PF4 [140], CXCL13 [141] bind FGF2, inhibiting its interaction with HSPGs and FGFR1, FGF2 internalization, mitogenic and/or anti-apoptotic activity of cultured ECs and angiogenesis *in vivo*.

Several peptides derived from FGF2, FGFRs or the natural FGF2-binders described above have been demonstrated to exert an inhibitory activity on the FGF2/FGFR system (Table 4). Among the more than 80 FGF2 antagonist peptides so far described, only a few of them have been identify or characterized by SPR analysis. Nevertheless, these studies show the potentiality of this technology for a systematic identification of FGF2 inhibitors.

Regarding peptides that bind and sequester FGF2, SPR has been exploited to demonstrate that the FGF2-derived peptide KRTGQYKLC inhibits FGF2/FGFR1 interaction by binding to FGF2 itself rather than to FGFR [74].

Regarding peptides that bind and mask FGF2 receptors, a 16-mer oligopeptide with no sequence homology to FGF2 but endowed with conformational similarity to its putative receptor-binding domain was demonstrated to effectively bind to FGFR1 immobilized to a SPR sensorchip [82]. Also, the high-through-put feature of SPR allowed a systematic study of the binding of up to 10 FGF-derived peptides (named dekafins) to the D2–D3 domain of both FGFR1-IIIc and FGFR2-IIIb [28]. Also, SPR has been used to demonstrate that endostatin binds to HSPGs, suggesting its angiostatic activity may depend, at least in part, on its capacity to mask these receptor to FGF2 [81]. Finally, SPR has been used to analyze large panels of TSP-derived [59] or PTX3-derived peptides [92].

**Figure 5.**
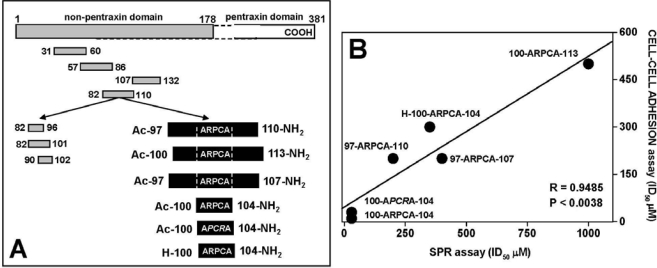
FGF2-antagonist activity of PTX3-derived peptides. A) Schematic representation of the peptides (in grey) spanning the N-terminal domain of PTX3 utilized for the identification of the amino acid sequence 97–110 as the FGF2-binding domain in PTX3 [92]. The synthetic peptides based on the PTX3(97–110) sequence and used for the analysis shown in panel B are reported in black. B) Relationship between the potency of the peptides to inhibit FGF2/FGFR1-IIIc interaction in a SPR assay and HSPG/FGF2/FGFR1 ternary complex formation in a FGF2-dependent CCA assay [expressed as the concentration of peptide required to obtain 50% inhibition (ID_50_)]. (Data from both the assays were the mean of three independent experiments, performed in duplicate for CCA assay).

In our previous work, by assaying a large panel of synthetic peptides encompassing the N-terminal extension of human PTX3 we were able to identify the FGF2-binding domain of PTX3 in the amino acid sequence 97–110 [92,138]. More recently, a series of synthetic peptides based on amino acid sequence PTX3(97–110) and containing different mutations in the Ala-Arg-Pro-Cys-Ala (ARPCA) motif (Figure 5A) were assessed for their FGF2-binding and antiangiogenic activity *in vitro* and *in vivo* [169]. As a preliminary experiment, the peptides were tested for their capacity to prevent the binding of FGF2 to FGFR1-IIIc (D1–D3) immobilized to a SPR sensorchip. Interestingly, a significant correlation was found between the capacity of these peptides to inhibit FGF2/FGFR1-IIIc interaction in the SPR-based assay and their ability to prevent the formation of a productive HSPG/FGF2/FGFR1 ternary complex in the FGF2-dependent cell-cell adhesion assay described above (Figure 5B).

### 4.2. Anti-FGF2 Polyanionic Heparin-Like Molecules

Free heparin sequesters FGF2 in the extracellular environment, thus exerting an antiangiogenic effect. However, mainly due to its anticoagulant activity and binding aspecificity, unmodified heparin cannot be used as an antiangiogenic drug [127]. This started a series of researches aimed at identifying non-anticoagulant heparin analogous and polyanionic compounds endowed with a more specific FGF2 antagonist activity (reviewed in [7,130,170]). In this context, our laboratory has been involved in the study of a powerful and versatile class of heparin-like molecules originated by chemical and enzymatic modifications of the polysaccharide K5 (reviewed in [171]). K5 is produced by *E. coli* and has the same structure of the biosynthetic precursor of heparin/HS. This allows the chemical synthesis of novel heparin-like K5 derivatives characterized by different patterns of sulfation and chemical structure and endowed with a significant antiangiogenic capacity but devoid of anticoagulant activity [172].

**Figure 6.**
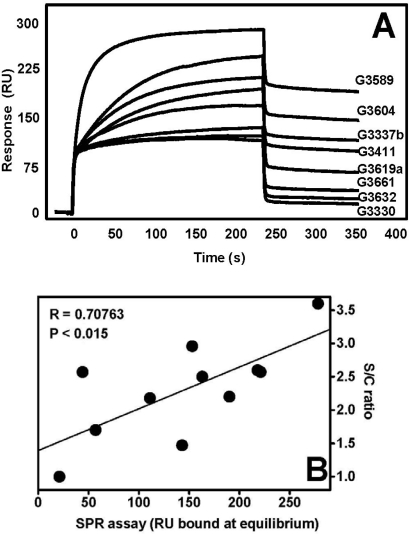
SPR runs of FGF2/K5 derivative interaction. FGF2 was immobilized to a sensorchip as described in [133]. Immobilized BSA was used as control and for blank subtraction. The various K5 derivatives (30 nM) were injected onto the FGF2- or BSA-coated sensor chips for four minutes and washed until full dissociation. After each run, the sensor chips were regenerated by a 2 M NaCl pulse [133]. A) Blank-subtracted sensorgram overlay showing the binding of K5 derivatives to sensorchip-immobilized FGF2. B) Plot of the blank-subtracted values of the SPR response at equilibrium for equimolar concentrations of each K5 derivative *versus* its sulfate/carboxyl (S/C) ratio. The SPR responses at equilibrium are taken from a single experiment out of three giving consistent results.

On this basis, we decided to assess whether SPR screening of K5 derivative/FGF2 binding might provide a valuable hint for structure-function correlation studies and might have a predictive value for the antiangiogenic potential of the heparin-like compound under test. To this purpose, in a first set of experiments we immobilized FGF2 to a SPR sensorchip and we used this assay to evaluate the individual binding capacity to FGF2 of a large panel of K5 derivatives differing for sulfation degree and chemical structure. Binding to FGF2 occurs through the negatively charged sulfated groups of the GAG [112]. Accordingly, we found a significant correlation between the degree of sulfation of K5 derivatives [expressed as sulfate/carboxyl group (S/C) ratio] and their capacity to bind FGF2 immobilized to the sensorchip [measured as the amount of K5 derivative that binds surface-immobilized FGF2 at the equilibrium] (Figure 6).

It is worth noticing that we intentionally avoided using in this analysis the binding affinities evaluated from the SPR data. The reason is that the state-of-the-art physicochemical models available for their evaluation rely on the approximations of bimolecular interactions described by the Scatchard’s equation [173,174] that are definitively too tight for the molecular system under evaluation. Indeed, the specific binding to FGF2 dependents not only on the overall sulfation degree of GAGs but also on the presence and number of definite sequences of sulfated groups within their backbone structure and may involve mechanisms of cooperative interaction [175]. This point reflects a pivotal basic aspect of the discussion about reliability and accuracy of binding parameters evaluated by SPR (and by solid-phase bioassays in general) and about their actual or presumed difference with the parameters evaluated in free solution and in cell-based assays (see below for a further discussion about this crucial point).

**Figure 7.**
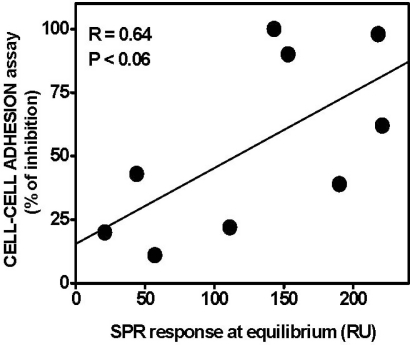
Correlation between SPR-generated binding parameters for the interaction of FGF2 with K5 derivatives and their antiangiogenic potential. SPR response at equilibrium of K5 derivatives to immobilized FGF2 were plotted *versus* the capacity of the FGF2 antagonist to inhibit HSPG/FGF2/FGFR1 ternary complex formation in the FGF2-dependent CCA assay (expressed as % of inhibition when the compounds were tested at the dose of 10 μg/mL). The data from CCA assays are the mean value from three different experiments performed in duplicate. The SPR responses at equilibrium are taken from a single experiment out of three giving consistent results.

In a second set of experiments, the K5 derivatives were assayed for their capacity to prevent the formation of a productive HSPG/FGF2/FGFR1 ternary complex in the FGF2-dependent cell-cell adhesion assay described above. Although with low statistical significance, a correlation trend appears to exist between the capacity of K5 derivatives to inhibit FGF2-dependent HSPG/FGF2/FGFR1 ternary complex formation and their ability to bind immobilized FGF2 (Figure 7).

These data support the hypothesis that SPR-generated parameters obtained from the analysis of the direct interaction of FGF2 with heparin-like polyanionic compounds may provide useful hints about the structure/function relationship of FGF2 antagonists. However, as shown in Figure 7, it is apparent that some K5 derivatives endowed with a very similar FGF2-binding capacity may show a quite different ability to prevent HSPG/FGF2/FGFR1 ternary complex formation and *vice versa*. This may be due to the capacity of some K5 derivatives to interfere directly with FGF2/FGFR1 interaction rather than with FGF2/HSPG interaction, calling for additional SPR experiments with sensorchip-immobilized FGFR1 (see Figure 5).

**Figure 8.**
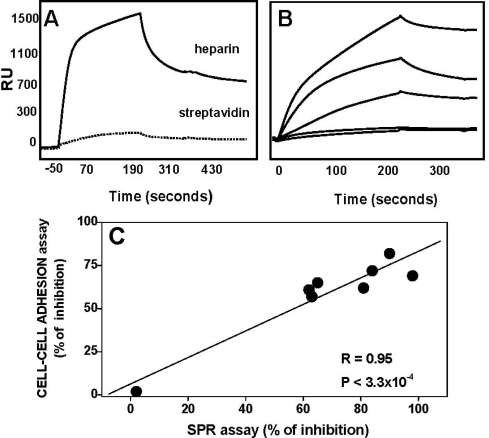
Capacity of LMW-K5 derivatives to prevent FGF2/heparin interaction in a SPR-based assay A) Heparin was biotinylated at its reducing end and immobilized onto a sensorchip previously activated with streptavidin. A streptavidin-activated sensorchip without biotinylated heparin was used as a negative control. FGF2 (260 nM) was injected over heparin-coated (straight lane) and control (dotted lane) sensor chips for four minutes and washed until dissociation was observed. After each run, the sensor chips were regenerated by a 2 M NaCl pulse. For further details see [94]. The response (in RU) was recorded as a function of time. Adapted from [94]. B) Sensorgram overlay showing the binding of FGF2 to immobilized heparin in the presence of increasing concentrations of free heparin. C) Different LMW-K5 derivatives were evaluated for their capacity to inhibit FGF2/heparin interaction in the SPR assay (expressed as % of inhibition when the compounds were tested at the dose of 300 nM) and to prevent HSPG/FGF2/FGFR1 ternary complex formation in the FGF2-dependent CCA assay (expressed as % of inhibition when the compounds were tested at the dose of 200 nM). Data from both CCA and SPR assays were the mean of three independent experiments (performed in duplicate for CCA assay).

As described above, FGF2 binds to HSPGs anchored to the surface of ECs. In order to more closely mimic the biological environment in which FGF2 antagonists may act, we exploited a second SPR experimental model in which heparin was immobilized to a SPR sensorchip and FGF2 was used as an analyte were performed [94]. FGF2 binds to immobilized heparin in a specific (Figure 8A) and dose-dependent manner (data not shown). Evaluation of the kinetic parameters of the interaction revealed that this occurs with parameters [K_on_ = 9.0 × 10^3^ M^−1^ s^−1^, K_off_ = 3.8 × 10^−4^ s^−1^, K_d_ = 42.5 nM] [94], consistent with previous measurements of the affinity of interaction of FGF2 with EC-associated HSPGs [112] (see also Table 2), further validating SPR analysis as a surrogate model for more classical cell culture-binding experiments. The binding of FGF2 to immobilized heparin was inhibited when the growth factor was co-injected on the sensorchip in the presence of increasing concentrations of free heparin (Figure 8B), thus confirming the possibility to use this SPR-based assay for the screening of polyanionic heparin-like FGF2 antagonists.

On this basis, a series of low molecular weight (LMW) K5 derivatives were evaluated for their capacity to prevent the binding of FGF2 to immobilized heparin and the SPR-generated data were compared to the data obtained when the same compounds were tested for the capacity to inhibit the formation of the HSPG/FGF2/FGFR1 ternary complex in the cell-cell adhesion assay described above. A clear correlation, exists between the results provided by the two assays (Figure 8C). This is in fair agreement with the results shown in Figure 7, further supporting the possibility to use SPR-based assays for the screening of polyanionic heparin-like FGF2 antagonists.

Interestingly, a similar correlation was found also when the SPR-generated data were challenged against the results obtained when the LMW-K5 derivatives were evaluated for their capacity to inhibit FGF2-dependent EC proliferation and sprouting, two widely diffused angiogenesis-related assays (for further details, see [94]). Finally, those compounds that turned out to be efficient FGF2 antagonists in the SPR assay showed a significant capacity to inhibit FGF2-angiogenesis *in vivo* in the chick embryo chorionallantoic membrane model [94]. These observations strongly support the exploitation of SPR measurements as a first-lane screening predictive of antiangiogenic activity.

The identification of lead compound(s) from a large library is only the first step in the development of effective antiangiogenic drugs. Indeed, lead compounds must be properly modified to increase their efficiency, specificity and safety. Also, this phase of the research calls for affordable and reliable systems that allow the real-time monitoring of the structure-function modifications.

## 5. Understanding the Surface-Confined Molecular Recognition: towards an Integrated Biosensing Strategy

As introduced in Section 2, SPR has been exploited to demonstrate and/or characterize the binding of AGFs with their interactors. However, as it emerges from Table 1 and 2, evaluation of kinetic and thermodynamic parameters by SPR experiments and data from different laboratories often provide significantly different results. According to the revised literature this may origin from various “biosensing” as well as “molecular” reasons, including:

- suboptimal data and/or over-interpreted results. A long list of these flaws, that are outside the target of this review, has been reported in details in the survey of commercial optical biosensor literature series [15–23]. It is important to note that a broad and detailed array of “guidelines” for the interpretation of SPR results is reported in these reviews. If correctly applied, these guidelines would reduce by a great extent the misinterpretations of SPR data, with great benefits for SPR studies in general and angiogenesis in particular.
- “lead up” or “lead down” experiments (e.g., the alternative choice to immobilize the AGF or its receptor onto the sensorchip surface). According to the state-of-the-art theory, the alternative choice of immobilization should lead to the same binding parameters. However, many “immobilization-driven” artefacts may affect the binding results under the different experimental conditions. Among them, mass transport effect may greatly (and differently) impact the lead up or lead down experiments. The comparison among binding data obtained at the solid-solution interface and in bulk solution may provide a valuable help in the interpretation of the results.
- different immobilization procedures that can mask or alter the accessibility of binding sites present in the ligand molecule;
- the use of different molecular forms of the ligand and/or of the analyte (e.g., different receptor isoforms, different AGF variants, GAGs of different chemical structure and charge).

Focusing on the “biosensing” issues, two intertwined actions are desirable in order to take a full advantage of SPR and other biosensor-based technologies for a better understanding of the molecular interactions during the angiogenesis process, as well as in other biological processes. From one hand, integrated approaches grounded on the combination of different biosensing techniques based on complementary transduction principles should be implemented. On the other hand, a deeper knowledge of ligand-receptor interactions confined at solid-solution interfaces may provide the basis for a better understanding of those interactions occurring at the (subtler) biological interfaces.

SPR spectroscopy ultimately relies on measuring the variation of the RI at the solid-solution interface that hosts the recognition event. However, any species adsorbed at the interface within few hundreds of nanometers can sort a variation of the RI and in turn generate a detection signal. Therefore, interpretation of SPR data might result problematic under not well-controlled conditions, as it is the case for non-specific adsorption, ligand-induced conformational changes, or multimeric and allosteric interactions. Also, SPR might present difficulties when utilized with low molecular weight analytes, since binding might not sort a measurable RI change. To overcome these problems, SPR experiments might be complemented by replicate experiments performed with label-free biosensors that feature alternative transduction mechanisms, such as quartz crystal microbalances that feature mass-based transduction, nanomechanical microcantilevers that feature mass- or energy-based transduction, electrochemical impedance spectroscopy, or isothermal titration (nano)calorimetry. All of these alternative techniques present advantages as well as drawbacks with respect to SPR.

Integration of different biosensors [176] will be more effective when an adequate understanding and control of ligand-receptor interactions confined at solid-solution interfaces and of the confining environment (including immobilization of the biomolecules onto the solid-phase) will be achieved On this subject, research is at the beginning and the literature somehow contradictory, pointing to substantial [176] or negligible [17,19] thermodynamic difference between the two systems. Basically, “surface effects” are recognized and qualitative *caveat* and solutions have been proposed. A throughout, updated, guideline for good practices can be found in the above cited reviews about commercial optical biosensor literature [15–23], in ref. [177] and, generally, in the application-notes supplied by the instrument dealers.

An alternative, promising evolution path is marked down by the development of a thermodynamic framework able to accurately describe ligand-receptor interactions confined at solid-solution interfaces [178,179].

## 6. Conclusions

Angiogenesis is the process of generating new capillary blood vessels. Uncontrolled neovascularization is observed in tumor growth and in angioproliferative diseases [2]. Tumors cannot grow larger than a few square millimetres as a mass unless a new blood supply is induced [1]. Hence the control of the neovascularization process may affect tumor growth and represent a novel approach to angiogenesis-dependent disease therapy, including neoplasia [180]. A complex molecular “interactome” due to the cross-talk among cell surface receptors, ECM components, and free AGFs appears to modulate the angiogenic balance in normal and pathological settings [7]. In this context, SPR can be usefully exploited to demonstrate and/or characterize the interaction of AGFs with their interactors and for the screening and characterization of antiangiogenic compounds.

SPR is a widely used powerful technology to study macromolecular interaction. Originally released in 1991, it has been used and cited in up to 10,000 papers in the last 12 years. Nevertheless, SPR has been sparingly employed in the study of angiogenesis-related molecules. In effect, a PubMed search on SPR and angiogenesis yielded only 80 papers (published from 1993 to date), 50% of them being devoted to the study of the molecular bases of the interaction of angiogenic growth factors with their receptors and the remaining ones to the exploitation of SPR for the identification of antiangiogenic compounds. Thus, the full potential of SPR in the study of angiogenesis remains largely unexploited.

Here, using the FGF2/FGFR1 system as a prototypic angiogenesis-related target, we have evidenced how SPR-based assays can be successfully used in the search and development of antiangiogenic compounds belonging to different classes of compounds, like synthetic peptides and heparin-like molecules.

It must be pointed out that human tumors in advanced stages of growth, usually characterized by a high degree of vascularization, may express various AGFs, suggesting that tumor neovascularization may represent the result of the simultaneous action of different AGFs [181]. Therefore, “multivalent” compounds (able to bind different AGFs) may be highly effective in inhibiting angiogenesis and tumor progression *in vivo*. Relevant to this point, several AGFs are endowed with heparin-binding capacity and must interact with HSPGs receptors to exert their full angiogenic potential [7]. To this respect, SPR-based assays may be of great advantage. Large libraries of putative HSPGs-antagonist can be easily and rapidly screened against a panel of heparin-binding AGFs in search of possible multitarget compounds able to inhibit simultaneously the interaction of different AGFs with heparin/HSPGs immobilized to the sensorchip (Table 5).

Relevant to this point, SPR has been already exploited for the screening of large libraries of polyanionic compounds directed against FGF2 and FGF1 [93,182] or against a panel of up to 6–10 different heparin-binding cytokines [72,73].

A deeper understanding of ligand-receptor interactions supported by solid-phase assays and the integrated use of label-free biosensors based on complementary transduction principles will greatly improve our comprehension of the molecular interactions occurring during the angiogenesis process, paving the road for the discovery of novel antiangiogenic drugs.

## Figures and Tables

**Table 1.** SPR analysis of the interaction of AGFs with their signalling receptors. SPR was used to assess the kinetics of interaction between the free AGF (analyte) and the extracellular domain of the cognate signalling receptor immobilized to the sensor chip (ligand).

**AGF**	**Immobilized receptor**	**Binding parameters**			**Reference**
		**K_on_** (M^−1^ s^−1^)	**K_off_** (s^−1^)	**K_d_** (nM)	

**FGF2**	FGFR1-IIIc (D1–D3)	1.1 × 10^5^	1.1 × 10^−2^	99.0	[24]
	FGFR1-IIIc (D2–D3)	9.6 × 10^4^	5.9 × 10^−3^	62.0	[25] *
	FGFR1-IIIc (D2–D3)	9.6 × 10^4^	6.0 × 10^−3^	62.0	[26]*
	FGFR1-IIIc (D1–D3)	3.0 × 10^5^	1.4 × 10^−6^	0.005	[A. Bugatti,UD]
	FGFR2-IIIb	1.3 × 10^6^	6.5 × 10^−4^	0.5	[27]
	FGFR3-IIIc (D2–D3)	No binding	No binding	No binding	[26] *

	α_v_β_3_ integrin receptor	5.1 × 10^4^	3.3 × 10^−4^	6.5	[A. Bugatti,UD]*

**FGF1**	FGFR1-IIIc	2.4 × 10^6^	4.1 × 10^−3^	35.0	[28]
	FGFR1-IIIc (D2–D3)	NR	NR	0.03	[29]
	FGFR1-IIIc (D2–D3)	2.2 × 10^5^	3.0 × 10^−2^	136.0	[26]*
	FGFR2-IIIb (D2–D3)	1.4 × 10^5^	4.7 × 10^−3^	59.6	[28]
	FGFR2-IIIb	8.0 × 10^5^	6.4 × 10^−4^	0.8	[27]
	FGFR3-IIIc (D2–D3)	8.8 × 10^5^	2.0 × 10^−1^	230.0	[30] *
	FGFR3-IIIc (D2–D3)	8.8 × 10^5^	2.0 × 10^−1^	230.0	[26] *
	FGFR3-IIIc (D1–D3)	2.0 × 10^5^	1.8 × 10^−1^	916.0	[30] *

	α_v_β_3_ integrin receptor	NR	NR	1,100.0	[29]

**FGF4**	FGFR1-IIIc (D2–D3)	2.6 × 10^5^	4.3 × 10^−2^	165.0	[26] *
	FGFR2-IIIb	1.5 × 10^6^	6.1 × 10^−4^	0.42	[27]
	FGFR3-IIIc (D2–D3)	No binding	No binding	No binding	[26] *

**VEGF-A_165_**	VEGFR2/KDR	3.6 × 10^6^	1.3 × 10^−4^	0.037	[31]
**VEGF-A_165_**	VEGFR2/KDR	6.6 × 10^4^	1.3 × 10^−5^	0.19	[32] *
**VEGF-A_165_**	VEGFR2/KDR	8.4 × 10^4^	3.2 × 10^−5^	0.38	[33] *
**VEGF-A_165_**	VEGFR2/KDR	0.5–2.2 × 10^6^	2.0–4.0 × 10^−4^	0.2–0.6	[34]
**VEGF-A_165_**	VEGFR2/KDR	5.7 × 10^4^	2.3 × 10^−6^	0.041	[A. Bugatti, UD]
**VEGF-A_165_**	VEGFR1/Flt	4.0 × 10^6^	3.0 × 10^−5^	0.007	[35]
**VEGF-A_165_**	VEGFR1/Flt	5.7 × 10^5^	1.7 × 10^−5^	0.03	[33] *
	
**VEGF-A_165_**	Neuropilin-1	NR	NR	NR	[36]
**VEGF-A_165_**	Neuropilin-1	1–10 × 10^5^	1.0 × 10^−2^	2,000.0	[37]
**VEGF-A_189_**	Neuropilin-1	NR	NR	NR	[38]
**VEGF-A_121_**	Neuropilin-1	No binding	No binding	No binding	[36]
**VEGF-A_109_**	Neuropilin-1	No binding	No binding	No binding	[37]

**VEGF-C**	VEGFR1/Flt	NR	NR	NR	[38]
	VEGFR2/KDR	NR	NR	NR	
	VEGFR3	NR	NR	NR	

VEGF **	VEGFR1/Flt	8.7 × 10^5^	1.5 × 10^−5^	0.017	[33] *
	VEGFR2/KDR	4.2 × 10^4^	2.7 × 10^−4^	6.5	

**HGF**	c-MET	1.2 × 10^5^	1.1 × 10^−2^	90.0	[39]
	c-MET	3.0 × 10^4^	6.2 × 10^−3^	50.0	[40] *
	c-MET	NR	NR	NR	[40]
	c-MET	NR	NR	NR	[41] *

**HIV-Tat**	VEGFR2/KDR	1.7 × 10^5^	1.2 × 10^−5^	0.07	[A. Bugatti, UD]

	α_v_β_3_ integrin receptor	1.2 × 10^7^	3.8 × 10^−1^	32.0	[42] *

**PDGF-BB**	PDGFRα	8.3 × 10^3^	1.2 × 10^−3^	150.0	[43]
**PDGF-BB**	PDGFRβ	9.5 × 10^5^	1.5 × 10^−3^	1.6	
**PDGF-AA**	PDGFRα	1.1 × 10^5^	1.5 × 10^−3^	13.4	
**PDGF-AA**	PDGFRβ	3.5 × 10^3^	1.6 × 10^−3^	453.0	

* studies performed by immobilizing the AGF to the sensor chip and by using the free receptor as analyte.

** VEGF from venom gland of Taiwan habu. UD, unpublished data. NR, data not reported.

**Table 2.** SPR analysis of the interaction of AGFs with extracellular proteoglycans. Analyses were performed by using the free AGF as analyte and by immobilizing the indicated proteoglycan to the sensorchip.

**AGF**	**Immobilized proteoglycan**	**Binding parameters**			**Reference**
		**K_on_** (M^−1^ s^−1^)	**K_off_** (s^−1^)	**K_d_** (nM)	

**FGF2**	agrin	1.8 × 10^5^	4.6 × 10^−4^	2.5	[44]
	syndecan 1/4	1.6 × 10^7^	4.4 × 10^−2^	2.5	[45]
	HSPG	8.5 × 10^5^	1.3 × 10^−2^	14.7	[46]
	HSPG (perlecan)	NR	NR	NR	[47] *
	HSPG (glypican)	NR	NR	NR	[48]

	CSPG	7.7 × 10^5^	2.3 × 10^−2^	30.5	[49]
	CSPG	1.5 × 10^5^	2.0 × 10^−4^	12.7	[50]*
	CSPG	No binding	No binding	No binding	[45]

**FGF1**	HSPG (perlecan)	NR	NR	NR	[51]
	HSPG (perlecan)	NR	NR	NR	[52]

	CSPG	No binding	No binding	No binding	[49]

**FGF4**	HSPG	1.7 × 10^5^	1.5	NR	[46]

**VEGF-A_165_**	CSPG	7.0 × 10^5^	1.7 × 10^−2^	24.0	[49]

**MK**	CSPG	8.2 × 10^4^	8.9 × 10^−5^	1.5	[49]
	CSPG	1.3 × 10^4^	4.8 × 10^−3^	367.0	[45]

	HSPG (syndecan 1/4)	6.9 × 10^4^	1.7 × 10^−3^	25.9	[45]

**PTN**	CSPG	4.2 × 10^5^	7.4 × 10^−5^	0.2	[49]
	CSPG	6.6 × 10^3^	3.5 × 10^−2^	5210.0	[45]
	CSPG	2.0 × 10^6^	2.7 × 10^−4^	0.14	[53] *

	HSPG	7.6 × 10^5^	8.9 × 10^−3^	11.9	[45]

**HB-EGF**	CSPG	1.1 × 10^6^	9.1 × 10^−3^	10.0	[49]

**PDGF-BB**	HSPG	2.4 × 10^5^	7.8 × 10^−4^	3.0	[54]
**PDGF-AA**	HSPG	3.4 × 10^4^	7.8 × 10^−4^	23.0	

**PDGF-AA**	CSPG	8.5 × 10^4^	2.2 × 10^−3^	25.9	[50] *

* studies performed by immobilizing the AGF to the sensorchip and using the proteoglycan as analyte. When not specified, the proteoglycan species was not identified. CSPG, chondroitin-sulfate proteoglycan; HSPG, heparan-sulfate proteoglycan; MK, midkine; PTN, pleiotrophin; HB-EGF, heparin-binding epithelial growth factor; NR data not reported.

**Table 3.** SPR-based experimental models utilized to study AGF/receptor interactions and to identify AGF/receptor inhibitors.

**Experimental model**		**AGF (references)**
**AFG/receptor interaction**	**Ligand:** receptor **Analyte:** AGF	FGF2 [24,25,27,44–46,48,49]; FGF1 [27–29,49,51,52]; FGF4 [26,27,46,49]; VEGF [31,34–38,49]; MK, PTN [45,49]; HB-EGF [49]; HGF [39,40]; HIV-Tat [A. Bugatti, UD]; PDGF [54]
	**Ligand:** AGF **Analyte:** receptor	FGF2 [25,26,47,50]; FGF1 [26,30]; FGF4 [27]; VEGF [32,33]; PTN [53]; HIV-1 Tat [42]; HGF [40,41]; PDGF [50]
**AGF/inhibitor or receptor/inhibitor interactions**	**Ligand:** AGF **Analyte:** inhibitor	FGF2 [59]; FGF4[60]; VEGF [61–68]; HIV-Tat [69,70]; PDGF [71]
	**Ligand:** inhibitor **Analyte:** AGF	FGF1 [72,73]; FGF2 [25,59,72–75]; VEGF [72,73,76–79]; IL-8 [72]; PDGF [73]; HGF [73]
	**Ligand:** receptor **Analyte:** inhibitor	FGF1 [80]; FGF2 [74,81,82]; VEGF [61,66,68,83–87]; PDGF [43]; angiopoietin [86]
	**Ligand:** inhibitor **Analyte:** receptor	FGF1 [28]; FGF2 [74,88]; VEGF [89]
**competition experiments: inhibitor *vs* analyte**	**Ligand:** receptor **Analyte:** AGF	FGF1 [80]; FGF2 [48] [A. Bugatti, UD]; VEGF [34]; HB-EGF [49]; MK & PTN [45]
	**Ligand**: AGF binder (e.g., heparin) **Analyte:** AGF	FGF1 [90,91]; FGF2 [72,74,90–94]; FGF8 [A. Bugatti, UD]; HIV-Tat [70,95]; VEGF [91,96,97]; PDGF [98]

UD, unpublished data.

**Table 4.** Synthetic peptides endowed with FGF2-antagonist capacity.

**Protein of origin**	**Peptides**	**Target**	**References**
**FGF2**	FGF2(48–58) (FREG)	FGF2	[142]
	FGF2(38–61)	?	[143]
	FGF2(82–101)	?	[143]
	FGF2(119–126)	FGF2	[74]*
	FGF2-derived DGR-containing peptides (4 peptides studied)	?	[143]
	FGF2(68–77)	FGFR	[144]
	FGF2(24–68) (Peptide D)	FGFR	[145,146]
	FGF2(93–120) (Peptide N)	FGFR	[145]
	FGF2(106–115)	FGFR	[145,146]
	FGF2(103–146)	FGFR	[147]
	F2A4-K-NS	FGFR	[24]
**FGFs (β10-β11 loop)**	dekafins (homologous to the NCAM FGFR-binding region)	FGFR	[28]*
**FGF1**	FGF1(112–147) and related peptides	FGFR	[148]
	FGF1(99–108)	FGFR	[149]
	FGF1 mimetics (6 peptides studied)	FGFR	[150]
**FGF5**	FGF5(95–104) (peptide P3)	FGFR	[151]
**N-cadherin**	EDC4 mimetics (2 peptides studied)	FGFR	[150]
**PTX3 N-terminus**	PTX3(97–110) (and 28 related peptides)	FGF2	[92]*
**PF4**	PF4(47–70)	FGF2	[152]
**Myelin Basic Protein**	MBP(152–167)	FGFR	[153]
**TSP-1**	4N1K	?	[154]
	(type III repeats-derived peptides) (6 peptides studied)	FGF2	[59]*
**NCAM(681–695)**	FGL	FGFR	[155–158]
	FRM-10	FGFR	[156,158,159]
	FRM-10 cyclic	FGFR	[156,158,159]
	FRM-13	FGFR	[156,158,159]
	DekaCAM	FGFR	[28,156,158]
	BCL	FGFR	[156,158,160]
	Encamin A	FGFR	[156,161]
	Encamin C	FGFR	[156,161]
	Encamin E	FGFR	[156,161]
**Random phage epitope library screening**	Epitope sequence	FGFR	[162]
	FGF2(13–18)	FGFR	[162]
	FGF2(119–126)	FGFR	[162]
	FGF2(120–125)	FGFR	[162]
	Peptide P7 (hydrophobic)	FGF2	[163]
	C19 (3 peptides studied)	FGFR	[164–166]
	Peptide P2 (hydrophobic)	FGFR	[167]
**Molecular modelling**	16–24 mer peptides	FGFR	[82,168]*

?: target and/or mechanism of action not characterized.

* studied by SPR.

**Table 5.** Polyanionic heparin-like compounds investigated by SPR for their capacity to inhibit the binding of AGFs to heparin immobilized to the sensorchip.

**Heparin-like compounds**	**AGF**	**Reference**
K5 derivatives suramin analogs	FGF2	[94] [M. Presta, UD]
K5 derivatives	FGF8	[M. Presta, UD]
Glycol-split heparins phosphosulfomannan derivatives	VEGF	[96] [97]
pentosan polysulfate sulfonic acid polymers K5 derivatives	HIV-Tat	[95] [70] [A. Bugatti, UD]
partially digested CS*	HB-EGF	[49]

* Tested on immobilized CS. UD, unpublished data.
